# Beta-lactam plus macrolide treatment versus beta-lactam monotherapy for community-acquired pneumonia: a propensity score analysis using data from a multicenter prospective cohort study

**DOI:** 10.1186/s12879-025-12408-x

**Published:** 2025-12-29

**Authors:** Kei Nakashima, Masahiro Aoshima, Hiroki Matusi, Atsushi Shiraishi, Hiroyuki Ito, Motoi Suzuki, Konosuke Morimoto, Koya Ariyoshi, Adult Pneumonia Study Group – Japan

**Affiliations:** 1 Department of Pulmonology, Kameda Medical Center, 929 Higashi-cho, Kamogawa, Chiba 296-8602 Japan; 2 BAY FRONT CLINIC MinamiFunabashi, LaLa Terrace TOKYO-BAY 2F, 2-2 Wakamatsu, Funabashi-shi, Chiba 273-0013 Japan; 3 Department of Clinical Epidemiology and Health Economics, School of Public Health, The University of Tokyo, 7-3-1, Hongo, Bunkyo-ku, Tokyo 113-0033 Japan; 4 Clinical Research Support Office, Kameda Medical Center, 929 Higashi-cho, Kamogawa, Chiba 296-8602 Japan; 5 Emergency and Trauma Center, Kameda Medical Center, 929 Higashi-cho, Kamogawa, Chiba 296-8602 Japan; 6 Department of Clinical Medicine, Institute of Tropical Medicine, Nagasaki University, 1-12-4 Sakamoto, Nagasaki, 852-8523 Japan; 7 Department of Respiratory Infections, Institute of Tropical Medicine, Nagasaki University, 1-12-4 Sakamoto, Nagasaki, 852-8523 Japan; 8 Infectious Disease Surveillance Center, National Institute of Infectious Disease, 1- 23-1 Toyama, Shinjuku-ku, Tokyo, 162-8640 Japan

**Keywords:** Anti-bacterial agents, Beta-Lactams, Macrolides, Pneumonia, Propensity score

## Abstract

**Background:**

Community-acquired pneumonia (CAP) substantially contributes to mortality and morbidity globally, with beta-lactams being a primary therapeutic agent. The efficacy of adding macrolides to beta-lactams in CAP treatment remains controversial. Here, we evaluated whether beta-lactam plus macrolide treatment (BLM) is more effective than beta-lactam monotherapy (BL) for preventing CAP mortality.

**Methods:**

We performed a secondary data analysis of a multicenter prospective cohort study involving patients diagnosed with CAP at four institutions. We selected patients treated with either BLM or BL. The primary endpoint was the outcome at the end of the observation period (death or recovery). The secondary endpoints were the length of hospital stay and duration of antibiotic use. Multiple imputations with bootstrapping were used to address missing data. Background characteristics were adjusted via propensity score matching.

**Results:**

Of the 3,470 patients initially included in the study, 2,784 were analyzed; 306 received BLM and 2,478 received BL. The average observation period for the groups was 17.0 (± 18.4) and 24.0 days (± 24.6), respectively. After propensity score matching, mortality was similar between the groups (5.06% for BLM vs. 4.98% for BL; difference 0.00, 95% confidence interval [CI] − 3.73 to 3.71), as were recovery rates (91.79% for BLM vs. 91.69% for BL; difference 0.00, 95% CI − 4.48 to 4.82). In the subgroup analysis of patients with severe CAP, mortality was 12.00% for BLM vs. 13.33% for BL (difference 0.00, 95% CI − 20.00 to 16.13), and recovery rates were 82.86% vs. 83.33% (difference 0.00, 95% CI − 20.00 to 20.00).

**Conclusion:**

Similar outcomes were observed in the mortality and recovery rates between the BLM and BL groups among patients with CAP. Clinicians should thoughtfully weigh the benefits of BLM against the potential risks, including adverse effects and antimicrobial resistance, when managing patients with CAP.

**Trial registration:**

This study protocol was registered with the University Hospital Medical Information Network Clinical Trials Registry (UMIN-CTR), identifier UMIN000006909, on December 19, 2011.

**Supplementary Information:**

The online version contains supplementary material available at 10.1186/s12879-025-12408-x.

## Introduction

Community-acquired pneumonia (CAP) represents the most prevalent infectious cause of mortality globally, accounting for 2.6 million deaths per year, according to the report of World Health Organization [1]. CAP is associated with a 30-day all-cause risk-standardized mortality rate of 11.6% and readmission rate of 18.2% [2]. Beta-lactams, which primarily target bacterial pathogens such as *Streptococcus pneumoniae*, represent the principal therapeutic agents for CAP [3]. In empirical therapy for hospitalized patients or patients with comorbidities, macrolides are generally added to beta-lactams to treat infections of atypical pathogens, particularly severe CAP [3, 4].

The efficacy of adding macrolides to beta-lactams in reducing mortality in the treatment of CAP remains controversial [5, 6]. Several prospective and retrospective observational studies have reported that dual therapy involving beta-lactam plus macrolide treatment (BLM) reduces the 30-day and in-hospital mortality of hospitalized patients with CAP, particularly in those with severe disease [5, 7–9]. In contrast, data from randomized controlled trials (RCTs) have not shown a mortality benefit despite showing an improvement in early clinical response [10, 11]. An RCT demonstrated that patients in the BLM group exhibited a more favorable trend towards achieving clinical stability after 7 days of treatment than those in the beta-lactam monotherapy (BL) group. However, there were no differences in the 30- and 90-d mortality rates between the BLM and BL groups [11]. Moreover, a cluster RCT found that BL was not inferior to BLM for the treatment of CAP in patients admitted to non-ICU wards in terms of 90-d mortality [12]. Furthermore, a recent network meta-analysis of RCTs found no significant difference in efficacy between BLM and BL for CAP treatment [13]. Moreover, a recent RCT of patients with CAP and systemic inflammatory response syndrome showed that, compared with BL, BLM improved the early clinical response, but the 28- and 90-day mortality rates did not differ between the treatment groups [10].

Further contributing to this debate, a recent (2025) large, real-world observational study from the United Kingdom found that BLM was not associated with a reduction in mortality, even in patients with severe pneumonia [14]. This finding is consistent with the results from previous RCTs. The persistent controversy highlights an urgent need for additional high-quality, real-world evidence. Such evidence is crucial for inclusion in future meta-analyses to definitively resolve these clinical uncertainties [15].

Therefore, our objective was to assess the effectiveness of BLM compared with BL in reducing mortality across the entire spectrum of CAP, using data from a multicenter prospective cohort study with propensity score matching to control for differences in baseline patient characteristics between groups.

## Methods

### Study setting and population

We performed a secondary analysis of data from a multicenter prospective cohort study of patients with CAP collected by the Adult Pneumonia Study Group-Japan (APSG-J), utilizing propensity score analysis. The APSG-J study prospectively collected data on patients diagnosed with CAP from both outpatient and inpatient services at Ebetsu City Hospital, Kameda Medical Center, Chikamori Hospital, and Juzenkai Hospital between September 2011 and August 2014, using a study protocol previously described [16]. CAP was diagnosed when all the following criteria were met: patients (1) aged ≥ 15 years, (2) exhibited symptoms compatible with pneumonia, such as fever, cough, sputum production, pleuritic chest pain, and dyspnea; and (3) displayed new pulmonary infiltrates on chest X-ray images or CT scans consistent with pneumonia. Our analysis included all participants enrolled in APSG-J. Chest X-rays were performed within 24 h of admission, while CT scans were performed at the discretion of the attending physicians. The exclusion criteria were as follows: (1) patients did not receive antimicrobial agents; (2) patients who were initially treated solely with macrolides; and (3) patients who were initially treated with antibiotics other than beta-lactams or macrolides, as well as those who received antifungal agents, antituberculosis drugs, or antiviral agents. To specifically measure the effect of BLM compared to BL, we excluded patients receiving macrolide monotherapy or antimicrobial agents other than those in the two classes of interest.

The study was conducted in accordance with the Guideline for Ethical Aspects in Epidemiological Study (Ministry of Health, Labour and Welfare, Japan 2008). This study received approval from the review board of the Institute of Tropical Medicine at Nagasaki University and the review boards of Ebetsu City Hospital, Kameda Medical Center, Chikamori Hospital, and Juzenkai Hospital (registration no. 11063070). We obtained written informed consent from all conscious patients. Given that the study was observational and involved no invasive interventions or deviations from standard medical treatment, all the institutional review boards waived the requirement for written informed consent for a few unconscious patients. The study was registered with the University Hospital Medical Information Network (UMIN000006909).

### Treatment group definitions

Patients were categorized into two groups based on the initial treatment received: those who were started on a combination of beta-lactam and macrolide treatment (BLM group) and those treated with beta-lactam alone (BL group). Specifically, inclusion in the BLM group required that the patient received at least one dose of a macrolide antibiotic in conjunction with beta-lactam therapy. Although antibiotic dosing generally followed standard reference guidelines [17], the specific dosage regimens were determined by the attending physicians at each institution without standardization across study sites. The classification into these treatment groups occurred at the time of CAP diagnosis, and the observation period for outcomes commenced immediately thereafter.

### Outcome measures

The primary endpoint was the outcome at the end of the observation period (death or recovery). The end of the observation period was defined according to the patient’s clinical course. For hospitalized patients, follow-up was completed at the time of discharge. For patients whose pneumonia improved and were followed as outpatients, the date of the final outpatient visit related to CAP was considered the end of the observation period. If the final clinical outcome was unknown—such as in cases in which outpatient follow-up ended due to transfer to another hospital—follow-up was censored on the date of the most recent clinic visit. The outcomes were recorded at the end of the observation period, and included recovery, stable condition, deterioration, death, and transfer to another hospital. Death was defined as an in-hospital death from any cause. The secondary endpoints were the length of hospital stay and duration of antibiotic use.

### Microbiological test

Good quality sputum and blood specimens were collected on admission. If patients were unable to expectorate sputum, it was induced by inhalation of hypertonic saline soon after admission, and sputum was collected before antibiotic administration. Upon arrival at each hospital’s laboratory, clinical specimens were promptly processed. All sputum samples were subjected to semi-quantitative or quantitative cultures. In addition, these samples were analyzed at the Institute of Tropical Medicine, Nagasaki University, using in-house multiplex polymerase chain reaction (PCR) to detect a panel of bacterial and viral pathogens. This panel included three typical bacteria (*Streptococcus pneumoniae*,* Haemophilus influenzae*,* and Moraxella catarrhalis*), three atypical bacteria *(Mycoplasma pneumoniae*,* Chlamydophila pneumoniae*,* and Legionella pneumophila*), and 13 viruses (influenza A and B, respiratory syncytial virus, human metapneumovirus, parainfluenza virus types 1–4, rhinovirus, coronavirus 229E/OC43, adenovirus, and bocavirus). The specific primers and PCR protocols used have been detailed in previous studies [18, 19]. Commercial kits (Binax NOW; Alere Inc.) were also used to conduct urinary antigen tests for *Streptococcus pneumoniae* and *Legionella pneumophila*.

### Data collection

The APSG-J study prospectively collected the following clinical information: age, sex, registered hospital, treatment setting (outpatient or inpatient), history of hospitalization (hospitalization for more than 2 days within 3 months before CAP diagnosis), residing in a nursing home or convalescent facility, dialysis (within 30 days), preexisting comorbidities (diabetes, heart failure, liver disease, renal disease, dementia, malignancy, asthma, and chronic respiratory disease [chronic obstructive pulmonary disease and bronchiectasis]), prescribed drugs before admission (oral steroids, antacids, and sleep-inducing drugs), aspiration-associated factors (aspiration episodes, pre-existing impaired consciousness, neuromuscular disease, insertion or placement of devices (e.g., nasogastric tubes), cerebrovascular disease, and long-term bedridden status), vital signs at diagnosis (consciousness, heart rate, respiratory rate, systolic blood pressure, and body temperature), laboratory data at diagnosis (hematocrit, blood urea nitrogen, sodium, glucose, and albumin levels), chest X-ray findings (pleural effusion), microbiological test findings (culture, urinary antigen, and polymerase chain reaction [PCR] analysis), administered antibiotics, outcome at the end of the observation period, length of hospital stay, and duration of antibiotic use.

### Statistical analysis

Owing to the observational nature of this study, we used the available number of cases and did not perform any sample size calculations [15]. All statistical analyses were performed in R 4.3.0 for statistical computing (https://www.r-project.org/), with the add-on packages “tableone” for creating Table [20], “mice” for multiple imputation [21], “MatchIt” for propensity score matching [22], and “cmprsk” for survival analysis [23, 24]. All tests were two-tailed, and differences were considered statistically significant at *p* < 0.05. As there were several missing values, we used multiple imputation by employing chained equations to complement all missing values in the study variables and generated 50 datasets with five iterations. To calculate the 95% confidence interval (CI), we employed the bootstrap method described by Schomaker and Heumann to appropriately integrate uncertainty from both multiple imputation and resampling [25]. Specifically, we used “Method 1: MI Boot (pooled sample [PS])” from their study. Following this procedure, we generated 1000 bootstrap samples for each of the 50 imputed datasets. The estimates from all resulting samples were then pooled into a single distribution, and the lower and upper limit of the 95% CI was defined by the 2.5th and 97.5th percentiles of this pooled distribution. In addition, we conducted a sensitivity analysis by excluding all cases with missing data prior to imputation. After performing multiple imputations, no missing values remained, and the subsequent propensity score-matched analysis (described below) was conducted including all the relevant variables.

### Propensity score matching

A logistic regression analysis was used to estimate the propensity score to predict the use of BLM rather than BL from 34 pretreatment covariates, including age, sex, treatment setting (outpatient or inpatient), history of hospitalization (hospitalization for more than 2 days within 3 months before the diagnosis of CAP), residing in a nursing home or convalescent facility, dialysis (within 30 d before diagnosis), preexisting comorbidities (diabetes, heart failure, liver disease, renal disease, dementia, malignancy, asthma, and chronic respiratory disease [chronic obstructive pulmonary disease and bronchiectasis]), prescribed drugs before admission (oral steroids, antacid, and sleeping drugs), aspiration-associated factors (aspiration episodes, pre-existing impaired consciousness, neuromuscular disease, insertion or placement of devices (e.g., nasogastric tubes), cerebrovascular disease, and long-term bedridden status), vital signs at diagnosis (consciousness, heart rate, respiratory rate, systolic blood pressure, and body temperature), laboratory data at diagnosis (hematocrit, blood urea nitrogen, sodium, glucose, and albumin), and findings of chest X-ray (pleural effusion). We selected these as covariates because they are risk factors for antibiotic-resistant bacteria, prognostic factors, and risk factors for aspiration pneumonia [3, 26–28]. Propensity score matching selected participants pairwise on a 1:1 basis after all propensity scores across the imputed datasets were averaged and logit-transformed. The match caliper was set to standard deviation of the propensity score multiplied by 0.05. We used standardized mean differences (SMDs) of all variables included in the propensity score estimation to assess the match balance, and SMDs of < 0.1 were defined as appropriate match balance [29].

### Primary and secondary analyses

The primary endpoints were assessed by frequency in each group and absolute difference between the groups. The secondary endpoints were validated as continuous variables and absolute differences between the groups. A cumulative incidence curve of the primary endpoints (death or recovery) of patients was generated from one of the datasets after imputing missing values from the original dataset using multiple imputation [21]. To assess the efficacy of BLM in severe CAP treatment, a subgroup analysis was conducted on patients with a CURB-65 score of 3 or higher [30]. In addition, based on previous reports suggesting that BLM may be effective in treating pneumococcal and bacteremic pneumonia [31, 32], we conducted an exploratory subgroup analysis limited to patients with microbiologically confirmed non-atypical bacterial pneumonia. Microbiologically confirmed non-atypical bacterial pneumonia was defined by the presence of at least one of the following criteria: (1) a positive blood culture for a bacterial pathogen (excluding atypical pathogens) that could be the causative organism of pneumonia; (2) pleural fluid cultures yielding a bacterial pathogen other than atypical pathogens; or (3) a high-quality sputum sample (> 25 polymorphonuclear cells and < 10 epithelial cells per low-power field [total magnification ×100]) showing predominant growth of non-atypical bacterial pathogens in culture at ≥ 1 × 10⁶ CFU/mL, or a semiquantitative culture score of 3+; or (4) a positive pneumococcal urinary antigen test based on definitions used in previous studies [33, 34].

### Sensitivity analysis

To assess possible biases associated with multiple imputations, the primary outcome was reassessed using propensity score-matched analysis with the original dataset. In addition, we conducted a sensitivity analysis using a narrower caliper width of 0.01 for matching.

## Results

**Fig. 1:**
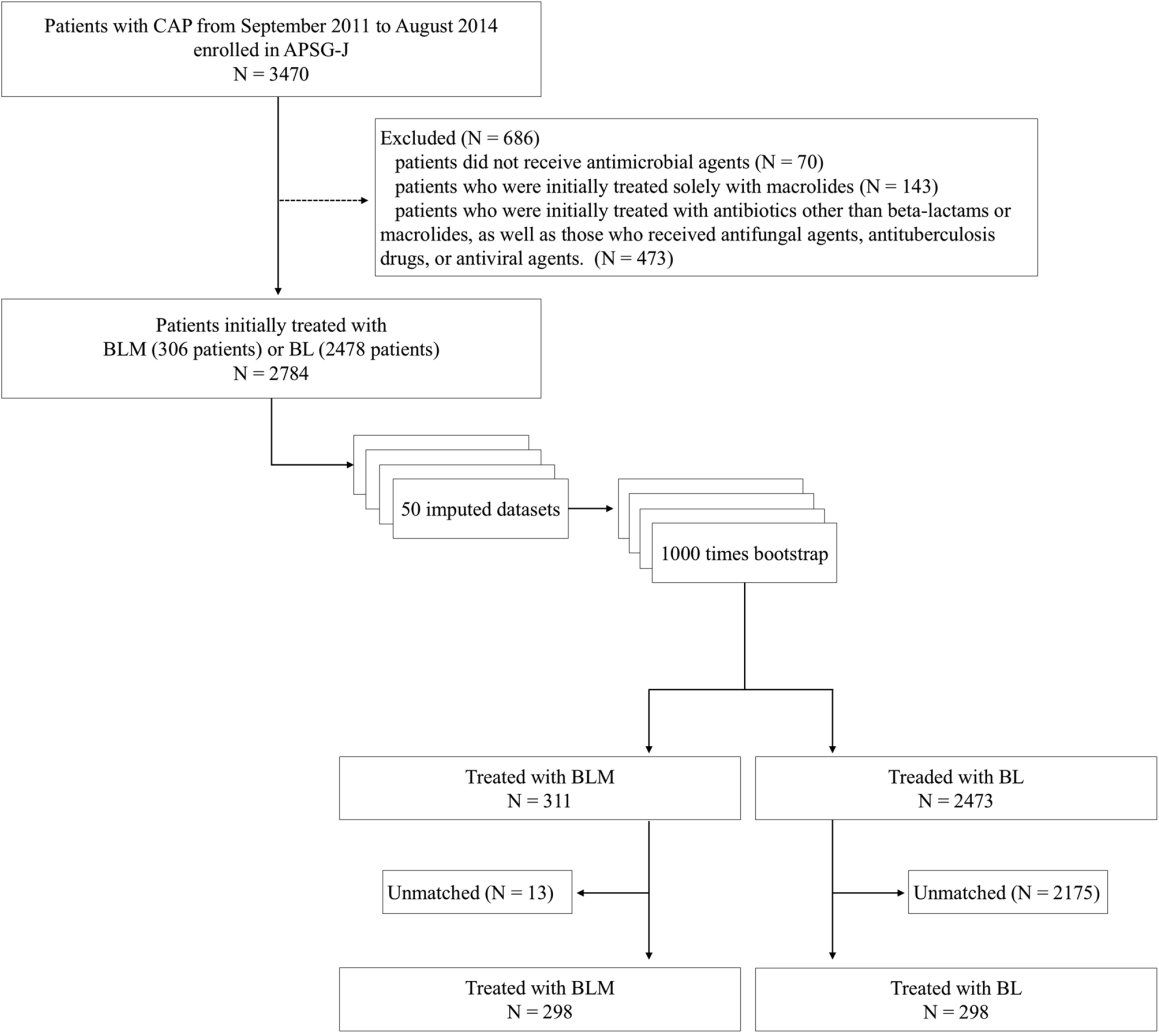
Flowchart of patient selection. In this analysis, 50 datasets were created using multiple imputation. Each dataset underwent 1,000 bootstrap resamplings, and one of the datasets was randomly selected to serve as a reference dataset. The imputation process resulted in slight variations in the sample sizes of the beta-lactam plus macrolide treatment (BLM) and beta-lactam monotherapy (BL) groups. (The original dataset had 306 patients and 2,478 patients in the BLM and BL groups, respectively, whereas the reference dataset generated using bootstrapping had 311 and 2,473 patients in the BLM and BL groups, respectively). These variations can be attributed to the differing imputed values across the datasets in the multiple imputation process and the variability introduced by the random sampling inherent in the bootstrap method. APSG-J, the Adult Pneumonia Study Group-Japan; BL, beta-lactam monotherapy; BLM, beta-lactam plus macrolide; CAP, community-acquired pneumonia

A flowchart of the patient selection process is shown in Fig. 1. Of the 3470 enrollees in the APSG-J study, 686 individuals were excluded. Subsequently, data of 2784 patients treated with BLM (306 patients) or BL (2478 patients) were analyzed.

Table 1 shows the pretreatment variables of patients with CAP in one of the datasets generated through bootstrapping of imputed datasets, before and after propensity score matching. SMDs < 0.1 and > 0.1 indicated balanced and unbalanced patient characteristics between groups, respectively. Before matching, the average observation periods for the two groups were 17.0 (± 18.4) days and 24.0 (± 24.6) days, respectively (*p* < 0.001). The mean age of the patients was 64.51 years (± 20.63) in the BLM group and 76.04 (± 15.08) years in the BL group, indicating that the patients in the BL group were older. Notably, 90% of the patients in the BLM group were enrolled at Kameda Medical Center. The most common comorbidities included diabetes mellitus, malignancy, and chronic obstructive pulmonary disease or bronchiectasis. Oral corticosteroid use was observed in 8.6% of the patients in the overall cohort. A history of aspiration was noted in 12.5% of patients in the BLM group and 28.6% of patients in the BL group, with a higher frequency in the latter group. Similarly, cerebrovascular disease was more prevalent in the BL group. Patients with a CURB-65 score of ≥ 3 were also more common in the BL group. After propensity score matching, 34 covariates were adjusted. Among these, 29 variables achieved an SMD of < 0.1, indicating balance, except for female sex, sleep-inducing drugs, pre-existing impaired consciousness, systemic blood pressure, and pleural effusion on chest X-ray. Furthermore, after propensity score matching, the observation period remained shorter in the BLM group than in the BL group (17.2 ± 18.6 days for BLM vs. 21.4 ± 24.0 days for BL; *p* = 0.017).

**Table 1:** Pretreatment variables of patients with community-acquired pneumonia in one of the datasets generated through bootstrapping of imputed datasets, before and after propensity score matching

Variable	Before matching			After matching		
	BLM	BL	SMD^a^	BLM	BL	SMD^a^
	*N* = 311	*N* = 2473		*N* = 298	*N* = 298	
Age, year	64.51 (20.63)	76.04 (15.08)	0.638	66.03 (19.50)	66.82 (19.71)	0.040
Female sex	142 (45.7)	952 (38.5)	0.145	134 (45.0)	117 (39.3)	0.116
Hospital			0.991			0.704
Chikamori Hospital	6 (1.9)	484 (19.6)		6 (2.0)	44 (14.8)	
Ebetsu City Hospital	25 (8.0)	344 (13.9)		24 (8.1)	34 (11.4)	
Juzenkai Hospital	0 (0.0)	339 (13.7)		0 (0.0)	24 (8.1)	
Kameda Medical Center	280 (90.0)	1306 (52.8)		268 (89.9)	196 (65.8)	
Treatment setting						
Outpatient	134 (43.1)	442 (17.9)	0.570	122 (40.9)	122 (40.9)	< 0.001
Risk of bacterial resistance						
Hospitalization for more than 2 days within 3 months	29 (9.3)	500 (20.2)	0.311	29 (9.7)	26 (8.7)	0.035
Residing in a nursing home or convalescent facility	15 (4.8)	443 (17.9)	0.421	15 (5.0)	13 (4.4)	0.032
Dialysis (within 30 days)	8 (2.6)	40 (1.6)	0.067	8 (2.7)	9 (3.0)	0.020
Comorbidity						
Diabetes mellitus	51 (16.4)	523 (21.1)	0.122	51 (17.1)	46 (15.4)	0.045
Heart failure	27 (8.7)	404 (16.3)	0.233	27 (9.1)	23 (7.7)	0.048
Liver disease	4 (1.3)	155 (6.3)	0.264	4 (1.3)	4 (1.3)	< 0.001
Renal disease	27 (8.7)	282 (11.4)	0.091	26 (8.7)	26 (8.7)	< 0.001
Dementia	10 (3.2)	424 (17.1)	0.473	10 (3.4)	9 (3.0)	0.019
Malignancy	35 (11.8)	510 (20.6)	0.258	35 (11.7)	35 (11.7)	< 0.001
Asthma	26 (8.5)	258 (10.4)	0.132	21 (7.0)	20 (6.7)	0.013
COPD or bronchiectasis	50 (16.4)	600 (24.3)	0.306	39 (13.1)	48 (16.1)	0.086
Medication						
Oral steroids	38 (12.5)	201 (8.1)	0.042	28 (9.4)	24 (8.1)	0.048
Antacids	82 (26.4)	746 (30.2)	0.084	79 (26.5)	83 (27.9)	0.030
Sleep-inducing drugs	29 (9.3)	315 (12.7)	0.109	29 (9.7)	17 (5.7)	0.151
Aspiration-associated risk factors						
Aspiration episodes	39 (12.5)	708 (28.6)	0.406	39 (13.1)	36 (12.1)	0.030
Pre-existing impaired consciousness	9 (2.9)	159 (6.4)	0.168	9 (3.0)	2 (0.7)	0.175
Neuromuscular diseases	8 (2.6)	199 (8.0)	0.246	8 (2.7)	7 (2.3)	0.021
Insertion or placement of devices (e.g., nasogastric tubes)	2 (0.6)	64 (2.6)	0.155	2 (0.7)	2 (2.3)	< 0.001
Cerebrovascular diseases	25 (8.0)	608 (24.6)	0.460	25 (8.4)	26 (8.7)	0.012
Long-term bedridden status	25 (8.0)	332 (13.4)	0.175	25 (8.4)	18 (6.0)	0.091
Vital signs at diagnosis						
Impaired consciousness	30 (9.6)	499 (20.2)	0.299	30 (10.1)	22 (7.4)	0.095
Heart rate, beats/minute	97.26 (17.90)	96.11 (20.39)	0.060	96.90 (17.82)	97.65 (19.36)	0.040
Respiratory rate, breaths/minute	21.91 (5.57)	22.52 (6.09)	0.105	21.96 (5.59)	22.49 (6.31)	0.090
Systolic blood pressure, mmHg	127.08 (23.46)	130.18 (25.79)	0.126	127.42 (23.57)	128.14 (19.36)	0.159
Body temperature, Celsius	37.50 (1.06)	37.45 (1.10)	0.050	37.50 (1.06)	37.47 (1.04)	0.031
Laboratory data at diagnosis						
Hematocrit, %	38.02 (5.46)	36.59 (5.89)	0.252	37.92 (5.53)	37.88 (5.72)	0.007
BUN, mg/dL	17.54 (12.99)	22.39 (15.57)	0.338	17.91 (13.12)	17.74 (10.60)	0.014
Na, mEq/L	137.80 (3.54)	137.62 (4.64)	0.044	137.76 (3.59)	137.73 (4.24)	0.006
Glucose, mg/dL	135.20 (59.95)	139.67 (59.01)	0.075	136.19 (60.60)	138.73 (60.59)	0.042
Albumin, g/dL	3.52 (0.55)	3.41 (0.56)	0.188	3.49 (0.54)	3.48 (0.59)	0.031
Pleural effusion on chest X-ray	11 (3.5)	186 (7.5)	0.175	11 (3.7)	6 (2.0)	0.101
CURB-65						
≥3	52 (16.7)	621 (25.1)	0.207	52 (17.4)	53 (17.8)	0.009
≥4	15 (4.8)	129 (5.2)	0.018	15 (5.0)	11 (3.7)	0.066

Data are presented as number (%) or mean (standard deviation)

^a^Propensity score matching was conducted using 34 variables: age, sex, treatment setting (outpatient or inpatient), history of hospitalization (hospitalization for more than 2 days within 3 months before the diagnosis of CAP), residing in a nursing home or convalescent facility, dialysis (within 30 days before diagnosis), preexisting comorbidities (diabetes, heart failure, liver disease, renal disease, dementia, malignancy, asthma, and chronic respiratory disease [COPD and bronchiectasis]), prescribed drugs before admission (oral steroids, antacids, and sleeping drugs), aspiration-associated factors (aspiration episodes, pre-existing impaired consciousness, neuromuscular disease, insertion or placement of devices (e.g., nasogastric tubes), cerebrovascular disease, and long-term bedridden status), vital signs at diagnosis (consciousness, heart rate, respiratory rate, systolic blood pressure, and body temperature), laboratory data at diagnosis (hematocrit, BUN, sodium, glucose, and albumin), and findings of chest X-ray (pleural effusion). An SMD of < 0.1 among the covariates was considered an appropriate match balance

BL, beta-lactam monotherapy; BLM, beta-lactam plus macrolide; BUN, blood urea nitrogen; CAP; community-acquired pneumonia; COPD, chronic obstructive pulmonary disease; SMD, standardized mean difference

Table 2 shows the microbiological characteristics in one of the datasets generated through bootstrapping of imputed datasets, before and after propensity score matching. Before matching, the most commonly identified bacteria in sputum cultures were *Streptococcus pneumoniae*, *Haemophilus influenzae*, methicillin-sensitive *Staphylococcus aureus*, *Pseudomonas aeruginosa*, *Moraxella catarrhalis*, and *Klebsiella pneumoniae*. Among the entire pre-matching cohort, *Mycoplasma pneumoniae* was the most commonly identified atypical pathogen (1.9%), followed by *Chlamydia pneumoniae* (0.2%). Among the urinary antigen tests, *Streptococcus pneumoniae* and *Legionella pneumophila* were positive in 9.2% and 0.1% (3 cases), respectively. The most frequently detected viruses on PCR testing of sputum samples, in descending order of prevalence were human rhinovirus, respiratory syncytial virus, influenza A virus, human parainfluenza virus type 3, and human metapneumovirus. After matching, bacterial pathogens, such as *Streptococcus pneumoniae*, *Haemophilus influenzae*, *Moraxella catarrhalis*, *Pseudomonas aeruginosa*, and *Escherichia coli*, were more frequently isolated in the BL group than in the BLM group. The number of patients with a positive pneumococcal urinary antigen test result was higher in the BL group. In contrast, positive blood cultures were more common in the BLM group. As for atypical pathogens, *Mycoplasma pneumoniae* PCR positivity was more frequent in the BLM group, and one patient in the BLM group tested positive for *L. pneumophila* on the urinary antigen test.

**Table 2:** Microbiological test findings, including culture and polymerase chain reaction analysis results, in one of the datasets generated through bootstrapping of imputed datasets, before and after propensity score matching

Causative pathogen	Before matching				After matching		
	BLM *N* = 311		BL *N* = 2473		BLM *N* = 298		BL *N* = 298
Sputum culture performed^a^	275 (88.4)		2305 (93.2)		267 (89.6)		268 (89.9)
* Streptococcus pneumoniae*	25 (8.0)		274 (11.1)		25 (8.4)		40 (13.4)
* Haemophilus influenzae*	32 (10.3)		246 (9.9)		31 (10.4)		35 (11.7)
Methicillin-sensitive *Staphylococcus aureus*	9 (2.9)		153 (6.2)		9 (3.0)		17 (5.7)
* Pseudomonas aeruginosa*	4 (1.3)		164 (6.6)		4 (1.3)		17 (5.7)
* Moraxella catarrhalis*	15 (4.8)		159 (6.4)		14 (4.7)		20 (6.7)
* Klebsiella pneumoniae*	4 (1.3)		141 (5.7)		3 (1.0)		1 (0.3)
Methicillin-resistant *Staphylococcus aureus*	4 (1.3)		78 (3.2)		4 (1.3)		6 (2.0)
* Escherichia coli*	2 (0.6)		72 (2.9)		2 (0.7)		10 (3.4)
* Klebsiella oxytoca*	0 (0.0)		14 (0.6)		0 (0.0)		4 (1.3)
* Serratia marcescens*	0 (0.0)		24 (1.0)		0 (0.0)		2 (0.7)
* Escherichia coli* (ESBL)	0 (0.0)		12 (0.5)		0 (0.0)		0 (0.0)
* Legionella pneumophila*	1 (0.3)		0 (0.0)		1 (0.3)		0 (0.0)
Urinary antigen of *Streptococcus pneumoniae* performed	194 (62.4)		1446 (78.6)		188 (63.1)		152 (51.0)
Positive	22 (7.1)		235 (9.5)		22 (7.4)		25 (8.4)
Urinary antigen of *Legionella pneumophila* performed	180 (57.9)		1109 (48.8)		176 (59.0)		123 (41.3)
Positive	1 (0.3)		2 (0.1)		1 (0.3)		0 (0.0)
Prompt antigen of influenza virus performed	39 (12.5)		467 (18.9)		39 (13.1)		53 (17.8)
positive	6 (1.9)		19 (0.8)		6 (2.0)		1 (0.3)
Sputum bacterial PCR performed	209 (67.2)		1943 (78.6)		205 (68.8)		206 (69.1)
* Streptococcus pneumoniae*	44 (14.1)		394 (15.9)		44 (14.8)		53 (17.8)
* Haemophilus influenzae*	51 (16.4)		280 (11.3)		50 (16.8)		46 (15.4)
* Moraxella catarrhalis*	47 (15.1)		222 (9.0)		46 (15.4)		21 (7.0)
* Mycoplasma pneumoniae*	22 (7.1)		31 (1.3)		20 (6.7)		8 (2.7)
* Chlamydia pneumoniae*	1 (0.3)		4 (0.2)		1 (0.3)		0 (0.0)
* Legionella pneumophila*	0 (0.0)		2 (0.1)		0 (0.0)		0 (0.0)
Sputum viral PCR performed	211 (67.8)		1943 (78.6)		207 (69.5)		205 (68.8)
Human rhinovirus	15 (4.8)		197 (8.0)		14 (4.7)		20 (6.7)
Respiratory syncytial virus	6 (1.9)		73 (3.0)		6 (2.0)		6 (2.0)
Influenza A	7 (2.3)		58 (2.3)		7 (2.3)		12 (4.0)
Human parainfluenza virus type 3	2 (0.6)		40 (1.6)		2 (0.7)		6 (2.0)
Human metapneumovirus	7 (2.3)		30 (1.2)		7 (2.3)		1 (0.3)
Human parainfluenza virus type 1	1 (0.3)		21 (0.8)		1 (0.3)		4 (1.3)
Influenza B	3 (1.0)		15 (0.6)		3 (1.0)		2 (0.7)
Human parainfluenza virus type 2	0 (0.0)		7 (0.3)		0 (0.0)		1 (0.3)
Human coronavirus (229E/OC43)	0 (0.0)		22 (0.9)		0 (0.0)		3 (1.0)
Human adenovirus	0 (0.0)		2 (0.1)		0 (0.0)		0 (0.0)
Human bocavirus	0 (0.0)		2 (0.1)		0 (0.0)		0 (0.0)
Blood culture performed^a^	193 (62.1)		1556 (62.9)		187 (62.8)		173 (58.1)
* Escherichia coli*	3 (1.0)		15 (0.6)		3 (1.0)		1 (0.3)
* Streptococcus pneumoniae*	3 (1.0)		13 (0.5)		3 (1.0)		0 (0.0)
Methicillin-sensitive *Staphylococcus aureus*	0 (0.0)		14 (0.6)		0 (0.0)		0 (0.0)
* Klebsiella pneumoniae*	0 (0.0)		12 (0.5)		0 (0.0)		1 (0.3)
* Haemophilus influenzae*	3 (1.3)		0 (0.0)		4 (1.3)		0 (0.0)
* Pseudomonas aeruginosa*	0 (0.0)		3 (0.1)		0 (0.0)		0 (0.0)
* Escherichia coli* (ESBL)	0 (0.0)		2 (0.1)		0 (0.0)		0 (0.0)
* Moraxella catarrhalis*	0 (0.0)		1 (0.0)		0 (0.0)		0 (0.0)
Methicillin-resistant *Staphylococcus aureus*	0 (0.0)		2 (0.1)		0 (0.0)		1 (0.3)

Data are presented as number (%)

^a^In the analysis of sputum and blood cultures, only pathogens assessed as clinically pertinent to pneumonia were reported. Bacteria of ambiguous clinical significance, uncommon bacteria, and organisms recognized as normal flora were excluded from the report

BLM, beta-lactam plus macrolide; BL, beta-lactam monotherapy; ESBL, extended spectrum beta-lactamase; PCR, polymerase chain reaction

Table 3 lists the antibiotics used by study participants in one of the datasets generated through bootstrapping of imputed datasets, before and after propensity score matching. Before matching, the most commonly used β-lactam antibiotics were, in descending order of frequency, ceftriaxone, ampicillin-sulbactam, piperacillin-tazobactam, amoxicillin, and amoxicillin-clavulanate. Azithromycin accounted for almost all macrolide use. After matching, ceftriaxone and piperacillin-tazobactam were more frequently used in the BLM group than in the BL group, whereas ampicillin-sulbactam was more frequently used in the BL group.

**Table 3:** Antibiotics used in this study participants in one of the datasets generated through bootstrapping of imputed datasets, before and after propensity score matching

Antibiotics	Before matching		After matching	
	BLM *N* = 311	BL *N* = 2473	BLM *N* = 298	BL *N* = 298
Beta-lactam^a^				
Ceftriaxone	139 (44.7)	794 (32.1)	132 (44.3)	102 (34.2)
Ampicillin-sulbactam	13 (4.2)	715 (28.9)	13 (4.4)	64 (21.5)
Piperacillin-tazobactam	46 (14.8)	450 (18.2)	46 (15.4)	35 (11.7)
Amoxicillin	62 (19.9)	207 (8.4)	58 (19.5)	45 (15.1)
Amoxicillin-clavulanate	57 (18.3)	204 (8.2)	53 (17.8)	45 (15.1)
Cefotaxime	10 (3.2)	79 (3.2)	10 (3.4)	11 (3.7)
Meropenem	0 (0)	45 (1.8)	0 (0.0)	3 (1.0)
Cefditoren-pivoxil	36 (11.6)	141 (5.7)	35 (11.7)	36 (12.1)
Cefepime	6 (1.9)	34 (1.4)	6 (2.0)	4 (1.3)
Ampicillin	1 (0.3)	19 (0.8)	1 (0.3)	2 (0.7)
Benzylpenicillin	2 (0.6)	5 (0.2)	2 (0.7)	0 (0.0)
Cefotiam	2 (0.6)	12 (0.5)	2 (0.7)	0 (0.0)
Piperacillin	0 (0)	9 (0.4)	0 (0.0)	1 (0.3)
Others	11 (3.5)	87 (3.5)	10 (3.4)	10 (3.4)
Macrolide				
Azithromycin	300 (96.5)		287 (96.3)	
Clarithromycin	8 (2.6)		8 (2.7)	
Erythromycin	3 (1.0)		3 (1.0)	

Data are presented as number (%)

^a^Owing to duplications, the total number of each column exceeded the number of patients in each group

BLM, beta-lactam plus macrolide; BL, beta-lactam monotherapy

**Fig. 2:**
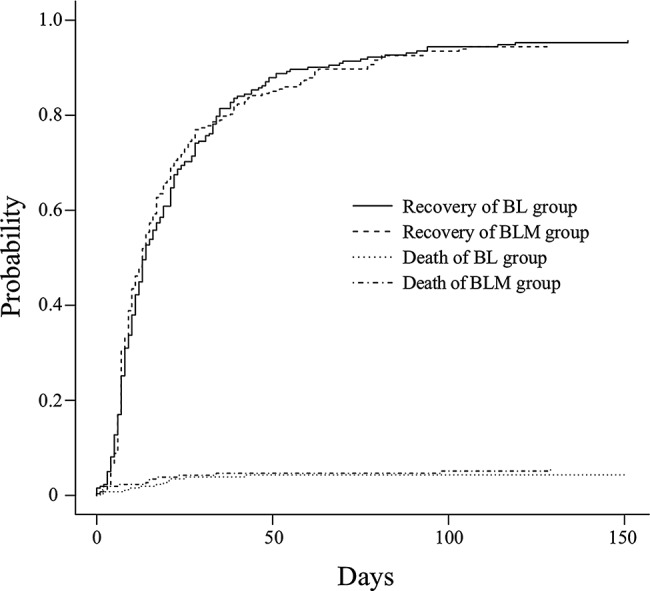
Cumulative incidence curve of the primary endpoints (death or recovery) of patients, generated from one of the datasets in which missing values in the original dataset were replaced with values imputed using multiple imputation. BL, beta-lactam monotherapy; BLM, beta-lactam plus macrolide

Table 4 presents the results for the primary and secondary endpoints. Regarding the primary endpoints, the death rate was 5.06% (95% CI 2.73%–7.78%) in the BLM group and 4.98% (95% CI 2.36%–8.21%) in the BL group. The absolute difference was 0.00% (95% CI − 3.73% to 3.71%). Similarly, the recovery rate was 91.79% (95% CI 88.43%–94.77%) in the BLM group and 91.69% (95% CI 87.73%–95.10%) in the BL group, with an absolute difference of 0.00% (95% CI − 4.48% to 4.82%). For the secondary endpoints, the length of hospital stay and duration of antibiotic treatment were similar between groups. Figure 2 shows the cumulative incidence curve of the primary endpoints (death or recovery) of patients, generated using one of the datasets in which missing values in the original dataset were replaced with values imputed using multiple imputation.

**Table 4:** Primary and secondary endpoints for the patients with community-acquired pneumonia treated with beta-lactam plus macrolide dual therapy and beta-lactam monotherapy

	BLM (*N* = 285)^a^	BL (*N* = 285)^a^	Absolute difference
Primary endpoints			
Death, %	5.06 (2.73–7.78)	4.98 (2.36–8.21)	0.00 (− 3.73 to 3.71)
Recovery, %	91.79 (88.43–94.77)	91.69 (87.73–95.10)	0.00 (− 4.48 to 4.82)
Secondary endpoints			
Duration of antibiotic treatment (days)	8.97 (8.46–9.51)	9.93 (9.01–17.10)	−0.99 (− 8.20 to 0.10)
Length of hospital stay (days)^b^	17.72 (15.29–20.50)	20.30 (17.31–24.09)	−2.59 (− 6.99 to 1.45)

Values in parentheses indicate the 95% CI

^a^N represents the point estimates derived from the median of the bootstrap results. The median and the 95% CI for N are 285 (253–318)

^b^Regarding the length of hospital stay, the number of cases was 166 (95%CI 140–192) in both the BLM and BL groups, as these endpoints were assessed exclusively in hospitalized patients

BLM, beta-lactam plus macrolide; BL, beta-lactam monotherapy; CI, confidence interval

Supplementary Table S1 presents the pretreatment variables of patients CAP in the subgroup with a CURB-65 score of 3 or higher from one of the bootstrapped imputed datasets, before and after propensity score matching. Despite a decrease in the number of cases and not all variables reaching SMD < 0.1, a statistical balance was attained for 17 of the 34 pretreatment covariates attempted to adjust, showing SMDs < 0.1, after matching. The results of the primary and secondary endpoint analyses are displayed in Table 5. No difference was observed in the death or recovery rates. Furthermore, the duration of antibiotic treatment and the length of hospital stay did not differ between the groups. Supplementary Table S2 shows the pretreatment variables of patients with CAP in the subgroup with microbiologically confirmed non-atypical bacterial pneumonia, using one of the bootstrapped imputed datasets, before and after propensity score matching. Although the number of cases decreased, 27 patients in each group were successfully matched. Supplementary Table S3 presents the primary and secondary endpoints of this subgroup. No differences were observed between the two groups in either primary or secondary outcomes.

**Table 5:** Primary and secondary endpoints for patients with a CURB-65 score of 3 or above who were treated for community-acquired pneumonia with beta-lactam plus macrolide dual therapy and beta-lactam monotherapy

	BLM (*N* = 29)^a^	BL (*N* = 29)^a^	Absolute difference
Primary endpoints			
Death, %	12.00 (0.00–25.71)	13.33 (0.00–29.41)	0.00 (− 20.00 to 16.13)
Recovery, %	82.86 (68.00–95.65)	83.33 (66.67–96.42)	0.00 (− 20.00 to 20.00)
Secondary endpoints			
Duration of antibiotic treatment (days)	9.62 (7.82–11.77)	10.52 (8.14–14.32)	−0.92 (− 5.07 to 2.30)
Length of hospital stay (days)^b^	24.06 (14.72–35.64)	23.57 (14.93–36.62)	0.28 (− 15.11 to 14.64)

Values in parentheses indicate the 95% CI

^a^N represents the point estimates derived from the median of the bootstrap results. The median and the 95% CI for N are 29 (16–44)

^b^Regarding the length of hospital stay, the number of cases was 21 (95%CI 10–34) in both the BLM and BL groups because these endpoints were assessed exclusively in hospitalized patients

BLM, beta-lactam plus macrolide; BL, beta-lactam monotherapy; CI, confidence interval

For the sensitivity analysis, we performed a complete case analysis. Supplementary Table S4 presents the pretreatment variables of patients with CAP in the original dataset including only complete cases with no missing values, before and after propensity score matching. In the complete case cohort, the proportion of outpatients was lower than that in the imputed datasets used in the primary analysis (236/1628 [14.5%] vs. 576/2784 [20.7%] before matching; 60/262 [22.9%] vs. 244/596 [40.9%] after matching). Supplementary Table S5 shows the primary and secondary endpoints. After matching, a balance was achieved between the groups for 25 of the 34 variables attempted for adjustment, with SMD < 0.1. The study outcomes were consistent with no differences in the primary endpoints of mortality and recovery rates. Similarly, no differences were observed in the secondary endpoints, namely, duration of antibiotic treatment and length of hospital stay. As an additional sensitivity analysis, we performed propensity score matching using a caliper width of 0.01. Supplementary Table S6 shows the pretreatment variables of patients with CAP in one of the datasets generated through bootstrapping of the imputed datasets, before and after matching using a caliper width of 0.01. Supplementary Table S7 shows the results for the primary and secondary endpoints. Consistent with the results of the main analysis, no differences were observed between the BLM and BL groups for any endpoint.

## Discussion

In this study, we compared the mortality and recovery rates between the BLM and BL groups in patients with CAP. Our findings indicated that the primary endpoints did not differ between the two treatments. Analysis of patients with a CURB-65 score of 3–5 showed that mortality and recovery rates were similar between the BLM and BL groups. A subgroup analysis limited to cases of microbiologically confirmed non-atypical bacterial CAP also revealed no differences between BLM and BL. Sensitivity analysis using complete case data and a narrower caliper of 0.01 showed no differences between the treatments.

The relevance of incorporating a macrolide with a beta-lactam in the management of CAP remains a contentious issue [5, 6]. Discordance is also observed among national guidelines. The American Thoracic Society and Infectious Diseases Society of America guidelines recommend the administration of beta-lactams and macrolides to outpatients with underlying diseases or to hospitalized patients [3]. Conversely, the United Kingdom and Japanese guidelines suggest prescribing beta-lactams and macrolides to patients with moderately severe CAP when atypical pathogens are suspected [4, 35]. For severe CAP, all guidelines advocate the use of beta-lactams and macrolides [3, 4, 35]. In this study, we did not detect an additional benefit of macrolides in the overall analysis or the subgroup analysis limited to cases with a CURB-65 of 3 or higher. Several previous observational studies—particularly those focusing on severe pneumonia—have reported that BLM therapy reduces mortality in patients with CAP, and these reports have served as a basis for the recommendations contained in the current guidelines [5, 7–9]. In contrast, several RCTs have not shown a mortality benefit of BLM therapy compared with BL therapy [10–12]. Two RCTs published in 2014 and 2015 did not demonstrate the superiority of BLM over BL in reducing mortality in the treatment of CAP [11, 12]. More recently, a 2024 RCT that examined patients with CAP along with systemic inflammatory response syndrome found that BLM enhanced the initial clinical response compared with BL [10]. However, it revealed no difference in mortality at both 28 and 90 days following treatment between the groups. The discrepancy between findings from observational studies and RCTs may be attributable to several factors, including unmeasured confounding in observational data, differences in patient characteristics, or variation in circulating pathogens across geographic regions [14]. Additionally, the lack of significant differences in mortality between groups in some RCTs may be attributable to insufficient statistical power due to limited sample sizes [10]. A recent large-scale observational study from the United Kingdom, published in 2025, used real-world data and adjusted for a broad range of patient characteristics and disease severity factors that could influence treatment selection; adjunctive macrolide therapy was not associated with improved mortality [14]. Our study showed similar mortality outcomes between the BLM and BL groups, consistent with the results of the United Kingdom study and those of RCTs [10–12, 14].

Macrolide therapy is associated with an increased risk of cardiovascular events and death [36, 37]. Macrolide combination regimens are effective in patients without cardiovascular diseases or patients with respiratory diseases and high leukocyte counts in the respiratory secretion [38]. Excessive use of macrolides is also a cause for concerns such as increasing emergence of macrolide-resistant bacteria [39]. The global importance of antimicrobial stewardship has been increasingly recognized. Avoiding unnecessary macrolide use is important from an antimicrobial stewardship perspective [40]. Therefore, healthcare professionals should understand both the advantages and disadvantages of macrolide use; make prescribing decisions accordingly, based on local epidemiological data and antibiogram profiles; and exercise caution when using macrolides in combination with beta-lactams for treating CAP.

Several potential mechanisms may account for the lack of an add-on effect of macrolides for reducing mortality in this study. First, the very low incidence of atypical pathogens in our cohort—including *Legionella pneumophila* (0.1%), *Mycoplasma pneumoniae* (1.9%), and *Chlamydia pneumoniae* (0.2%)—is likely to have attenuated any survival advantage conferred by empiric macrolide therapy. In previous observational studies reporting the effectiveness of BLM therapy, the proportion of *Mycoplasma pneumoniae* in the cohort was 2.8% [41], while the proportion of *Legionella species* ranged from 2.9% to 3.0% [41, 42]. A 2012 Cochrane review showed that broad atypical coverage (predominantly fluoroquinolone monotherapy) did not improve overall survival or clinical success in patients hospitalized for CAP, although clinical success was significantly higher in the subset with *Legionella pneumophila* infection [43]. In Japan, fluoroquinolones are often prescribed as soon as *Legionella pneumophila* is detected by urinary antigen testing; thereby limiting the number of cases of *Legionella pneumophila* infection available for inclusion in our study dataset. Second, previous observational studies showing the efficacy of BLM compared with BL may have been unadjusted for unknown confounding factors. A meta-analysis of RCTs in which the effects of unknown confounders were removed found no advantage of BLM over BL in terms of mortality [13]. In this study, after imputing missing values by employing multiple imputations followed by bootstrapping, we attempted to adjust 34 variables related to patient outcomes and treatment factors, including risk factors for antimicrobial resistance, prognosis, and aspiration-associated risk. We believe that we adjusted the covariates as much as possible. Third, the inclusion of cases of macrolide-resistant *Streptococcus pneumoniae* or *Mycoplasma pneumoniae* infection may have attenuated the effectiveness of BLM therapy. During the study period (2011–2014), many cases of macrolide-resistant *Streptococcus pneumoniae* and *Mycoplasma. pneumoniae* were reported in Japan [44–48]. Resistance mechanisms in *Streptococcus pneumoniae* include the presence of the *mef* gene, which promotes efflux of the antibiotic, and the *ermB* gene, which alters the antibiotic target site [47]. In *Mycoplasma pneumoniae*, macrolide resistance is primarily caused by point mutations in the ribosomal genes that encode the drug’s binding site [48]. These resistance patterns could have diminished the clinical effectiveness of the BLM regimen.

A strength of this study is that it used a multicenter prospective cohort with a large number of patients in a real-world setting. In addition to the overall analysis, we performed subgroup analysis for CAP patients with CURB-65 score ≥ 3 and conducted a complete case sensitivity analysis. Across all analyses, similar results were observed in the primary outcomes between the BLM and BL groups.

Future research should focus on specific patient subgroups, in which BLM therapy may be effective in reducing mortality. In our exploratory subgroup analysis of patients with microbiologically confirmed non-atypical bacterial pneumonia, mortality did not differ between the BLM and BL. However, this finding is inconclusive owing to the limited sample size. Previous studies have suggested that macrolides may exert beneficial anti-inflammatory and immunomodulatory effects [10], particularly in patients with systemic inflammatory responses or elevated inflammatory markers such as C-reactive protein or procalcitonin [10, 32]. Moreover, macrolides have been shown to be effective in patients with pneumococcal pneumonia and pneumonia with bacteremia [31, 32], highlighting the need to further investigate the potential benefit of macrolides in specific microbiologically defined subpopulations. As previously mentioned, underlying comorbidities—such as the presence or absence of cardiovascular or respiratory diseases—may also influence the effectiveness of BLM [38]. Therefore, further studies focusing on identifying specific subgroups based on inflammatory markers, microbiological findings, and underlying comorbidities are warranted. Clarifying these factors may contribute to the advancement of personalized treatment strategies for CAP. Until specific patient populations in which macrolides are effective are clearly established, the use of BLM should be limited to cases in which the use of BLM is recommended by current guidelines, such as patients with severe pneumonia or hospitalized patients, particularly those in whom atypical pathogens are suspected [3, 4, 35].

This study has several limitations. First, as an observational study, the potential for residual confounding from unmeasured variables remains despite the use of propensity score analysis, in contrast to RCTs. This concern is underscored by the fact that several variables did not achieve a SMD of less than 0.1 in the main analysis, including systolic blood pressure and pleural effusion on chest X-ray. These variables reflect disease severity. Furthermore, in the subset analysis of severely ill patients with a CURB-65 score of 3 or above, indicative of higher risk, we could successfully adjust only half of the 34 variables owing to the limited number of qualifying cases. Nevertheless, for the main analysis, our sensitivity analyses—complete-case analysis and propensity score matching with a narrower caliper of 0.01—were consistent with the primary analysis, indicating that any residual confounding had minimal impact on the overall findings. Second, the study may have been underpowered to exclude a clinically meaningful difference between the treatment groups, partly because of the lack of a priori sample size calculation and a low overall mortality rate (< 5%). This is highlighted in our primary analysis, where the 95% CI for the absolute difference in mortality (− 3.73% to 3.71%) crossed the 3% non-inferiority margin often considered clinically significant in trials of CAP [12, 49]. Thus, a modest, but clinically relevant, treatment effect cannot be ruled out. Nevertheless, publishing these findings is essential, as they will contribute valuable real-world evidence for inclusion in future meta-analyses, which can lead to more robust and precise conclusions [15]. Third, the generalizability of the findings may be limited. The study was conducted at four specific hospitals in Japan, with the majority of cases from a single center (Kameda Medical Center, as shown in Table 1), which could introduce bias from unmeasured institutional-level factors (e.g., patient management protocols). Moreover, a substantial number of patients (686 of 3,470) were excluded because they received other treatments, potentially creating a selection bias and limiting the applicability of our results to a broader patient population. Fourth, a methodological limitation concerns the interpretation of the observation period. A shorter duration was observed in the BLM group even after propensity score matching, which could suggest a faster clinical resolution. However, this finding should be interpreted with caution because the more direct secondary endpoints for hospitalized patients, including the length of hospital stay and duration of antibiotic use, did not show consistent differences across all analyses. This apparent discrepancy may be explained by the study design, as the observation period for non-hospitalized patients included the final outpatient visit. Moreover, because our data were collected at the hospital level rather than through individual patient follow-up, we were unable to comprehensively track all individual patient outcomes, which represents an inherent limitation in our outcome assessment. Therefore, further investigation is warranted to determine whether the addition of a macrolide to BL antibiotics conveys a true non-mortality benefit. Fifth, the observation period was relatively short, allowing only short-term outcomes to be assessed. Consequently, long-term endpoints, such as 90-day mortality, could not be evaluated. Sixth, data on antimicrobial susceptibility were not collected. Given the high prevalence of macrolide-resistant *Streptococcus pneumoniae* and *Mycoplasma pneumoniae* in the region [45–48], the inability to assess the impact of resistance patterns on treatment effectiveness is a significant limitation. Given these limitations, our results should be interpreted with caution.

## Conclusions

In this study, similar outcomes were observed in the mortality and recovery rates between the BLM and BL groups among patients with CAP, both in the overall population and in patients with a CURB-65 score of 3 or higher. Clinicians should thoughtfully weigh the benefits of BLM against the potential risks, including adverse effects and antimicrobial resistance, when managing patients with CAP. Further large-scale prospective studies are warranted to generate a hypothesis regarding whether BLM is superior in patients with certain baseline characteristics in terms of reducing mortality and to test it through an RCT.

## Supplementary Information

Below is the link to the electronic supplementary material.

Supplementary Material 1

## Data Availability

Data pertaining to this study will be available by the corresponding author upon reasonable request.
