# Supplementary material for: Beta-lactam plus macrolide treatment versus beta-lactam monotherapy for community-acquired pneumonia: a propensity score analysis using data from a multicenter prospective cohort study
Source: BMC Infect Dis. 2025 Dec 29;26:216. doi: 10.1186/s12879-025-12408-x (PMC12860014; doi:10.1186/s12879-025-12408-x)
Supplement: Supplementary file 1 — Supplementary Material 1 [file 12879_2025_12408_MOESM1_ESM.docx]

**Beta-lactam plus macrolide treatment versus beta-lactam monotherapy for community-acquired pneumonia: a propensity score analysis using data from a multicenter prospective study**

Supplementary Data

Supplementary Table S1 Pretreatment variables of patients with CAP in the subgroup with a CURB-65 score of 3 or higher from one of the bootstrapped imputed datasets, before and after propensity score matching

|  | Before matching | | |  | After matching | | |
| --- | --- | --- | --- | --- | --- | --- | --- |
| Variables | BLM  N = 52 | BL  N = 621 | SMD^a^ |  | BLM  N = 36 | BL  N = 36 | SMD^a^ |
| Age, years | 74.23 (11.10) | 80.08 (11.93) | 0.489 |  | 74.36 (10.33) | 78.08 (13.67) | 0.307 |
| Female sex | 26 (50.0) | 244 (39.3) | 0.217 |  | 14 (38.9) | 12 (33.3) | 0.116 |
| Hospital |  |  | 1.171 |  |  |  | 1.396 |
| Chikamori Hospital | 2 (3.8) | 137 (22.1) |  |  | 2 (5.6) | 11 (30.6) |  |
| Ebetsu City Hospital | 0 (0.0) | 78 (12.6) |  |  | 0 (0.0) | 4 (11.1) |  |
| Juzenkai Hospital | 0 (0.0) | 80 (12.9) |  |  | 0 (0.0) | 6 (16.7) |  |
| Kameda Medical Center | 50 (96.2) | 326 (52.5) |  |  | 34 (94.4) | 15 (41.7) |  |
| Treatment setting |  |  |  |  |  |  |  |
| Outpatient | 5 (9.6) | 54 (8.7) | 0.032 |  | 5 (13.9) | 4 (11.1) | 0.084 |
| Risk of bacterial resistance |  |  |  |  |  |  |  |
| Hospitalization for more than 2 days within 3 months | 9 (17.3) | 158 (25.4) | 0.199 |  | 9 (25.0) | 6 (16.7) | 0.206 |
| Residing in a nursing home or convalescent facility | 4 (7.7) | 164 (26.4) | 0.514 |  | 4 (11.1) | 4 (11.1) | <0.001 |
| Dialysis (within 30 days) | 4 (7.7) | 10 (1.6) | 0.292 |  | 0 (0.0) | 1 (2.8) | 0.239 |
| Comorbidity |  |  |  |  |  |  |  |
| Diabetes mellitus | 15 (28.8) | 151 (24.3) | 0.103 |  | 8 (22.2) | 6 (16.7) | 0.141 |
| Heart failure | 7 (13.5) | 131 (21.1) | 0.203 |  | 7 (19.4) | 3 (8.3) | 0.326 |
| Liver disease | 0 (0.0) | 44 (7.1) | 0.391 |  | 0 (0.0) | 0 (0.0) | <0.001 |
| Renal disease | 9 (17.3) | 81 (13.0) | 0.119 |  | 3 (8.3) | 6 (16.7) | 0.254 |
| Dementia | 4 (7.7) | 142 (22.9) | 0.431 |  | 4 (11.1) | 4 (11.1) | <0.001 |
| Malignancy | 9 (17.3) | 139 (22.4) | 0.128 |  | 7 (19.4) | 5 (13.9) | 0.149 |
| Asthma | 0 (0.0) | 63 (10.1) | 0.475 |  | 0 (0.0) | 0 (0.0) | <0.001 |
| COPD or bronchiectasis | 8 (15.4) | 148 (23.8) | 0.214 |  | 7 (19.4) | 6 (16.7) | 0.072 |
| Medication |  |  |  |  |  |  |  |
| Oral steroids | 10 (19.2) | 71 (11.4) | 0.218 |  | 4 (11.1) | 4 (11.1) | <0.001 |
| Antacids | 29 (55.8) | 220 (35.4) | 0.417 |  | 19 (52.8) | 15 (41.7) | 0.224 |
| Sleeping drugs | 7 (13.5) | 86 (13.8) | 0.011 |  | 5 (13.9) | 4 (11.1) | 0.084 |
| Aspiration-associated risk factors |  |  |  |  |  |  |  |
| Aspiration episodes | 10 (19.2) | 213 (34.3) | 0.345 |  | 8 (22.2) | 5 (13.9) | 0.218 |
| Pre-existing impaired consciousness | 7 (13.5) | 76 (12.2) | 0.037 |  | 4 (11.1) | 5 (13.9) | 0.084 |
| Neuromuscular diseases | 2 (3.8) | 64 (10.3) | 0.254 |  | 2 (5.6) | 3 (8.3) | 0.109 |
| Insertion or placement of devices (e.g., nasogastric tubes) | 1 (1.9) | 24 (3.9) | 0.116 |  | 0 (0.0) | 0 (0.0) | <0.001 |
| Cerebrovascular diseases | 11 (21.2) | 189 (30.4) | 0.213 |  | 5 (13.9) | 5 (13.9) | <0.001 |
| Long-term bedridden status | 6 (11.5) | 129 (20.8) | 0.429 |  | 2 (5.6) | 2 (5.6) | <0.001 |
| Vital signs at diagnosis |  |  |  |  |  |  |  |
| Impaired consciousness | 19 (36.5) | 312 (50.2) | 0.279 |  | 10 (27.8) | 15 (41.7) | 0.295 |
| Heart rate, beats/minute | 94.65 (19.59) | 98.79 (22.00) | 0.199 |  | 95.47 (20.97) | 97.14 (23.62) | 0.075 |
| Respiratory rate, breaths/minute | 25.75 (7.08) | 25.15 (7.30) | 0.083 |  | 24.28 (6.83) | 24.36 (6.46) | 0.013 |
| Systolic blood pressure, mmHg | 113.04 (24.69) | 120.29 (28.43) | 0.273 |  | 117.11 (24.58) | 120.11 (28.81) | 0.112 |
| Body temperature, Celsius | 37.56 (1.12) | 37.31 (1.29) | 0.202 |  | 37.42 (1.14) | 37.60 (1.16) | 0.157 |
| Laboratory data at diagnosis |  |  |  |  |  |  |  |
| Hematocrit, % | 34.49 (8.55) | 35.90 (6.41) | 0.186 |  | 34.18 (7.86) | 34.12 (6.75) | 0.007 |
| BUN, mg/dL | 27.01 (19.14) | 29.55 (17.41) | 0.139 |  | 24.43 (9.18) | 25.48 (9.88) | 0.109 |
| Na, mEq/L | 137.50 (5.50) | 138.27 (5.10) | 0.145 |  | 138.39 (2.80) | 138.39 (4.20) | <0.001 |
| Glucose, mg/dL | 143.85 (56.63) | 144.94 (66.40) | 0.018 |  | 141.11 (62.53) | 145.38 (51.52) | 0.078 |
| Albumin, g/dL | 3.20 (0.50) | 3.25 (0.63) | 0.089 |  | 3.29 (0.50) | 3.23 (0.54) | 0.122 |
| Pleural effusion on chest X-ray | 4 (7.7) | 51 (8.2) | 0.019 |  | 1 (2.8) | 2 (5.6) | 0.139 |
| CURB-65 |  |  |  |  |  |  |  |
| ≥4 | 15 (28.8) | 129 (20.8) | 0.188 |  | 8 (22.2) | 6 (16.7) | 0.141 |

Data are presented as number (%) or mean (standard deviation).

^a^Propensity score matching was conducted using the following 34 variables: age, sex, treatment setting (outpatient or inpatient), history of hospitalization (hospitalization for more than 2 d within 3 months before the diagnosis of CAP), residing in a nursing home or convalescent facility, dialysis (within 30 d before diagnosis), preexisting comorbidities (diabetes, heart failure, liver disease, renal disease, dementia, malignancy, asthma, and chronic respiratory disease [COPD and bronchiectasis]), prescribed drugs before admission (oral steroids, antacids, and sleeping drugs), aspiration-associated factors (aspiration episodes, pre-existing impaired consciousness, neuromuscular disease, insertion or placement of devices (e.g., nasogastric tubes), cerebrovascular disease, and long-term bedridden status), vital signs at diagnosis (consciousness, heart rate, respiratory rate, systolic blood pressure, and body temperature), laboratory data at diagnosis (hematocrit, BUN, sodium, glucose, and albumin), and findings of chest x-ray (pleural effusion). An SMD of <0.1 among the covariates was considered an appropriate match balance.

BL, beta-lactam monotherapy; BLM, beta-lactam plus macrolide; BUN, blood urea nitrogen; CAP; community-acquired pneumonia; COPD, chronic obstructive pulmonary disease; SMD, standardized mean difference

Supplementary Table S2 Pretreatment variables of patients with CAP in the subgroup with microbiologically confirmed non-atypical bacterial pneumonia from one of the bootstrapped imputed datasets, before and after propensity score matching

|  | Before matching | | |  | After matching | | |
| --- | --- | --- | --- | --- | --- | --- | --- |
| Variables | BLM  N = 44 | BL  N = 565 | SMD^a^ |  | BLM  N = 27 | BL  N = 27 | SMD^a^ |
| Age, years | 66.57 (15.87) | 76.43 (14.39) | 0.651 |  | 68.44 (14.38) | 70.74 (19.21) | 0.135 |
| Female sex | 22 (50.0) | 228 (40.4) | 0.195 |  | 13 (48.1) | 14 (51.9) | 0.074 |
| Hospital |  |  | 0.984 |  |  |  | 0.835 |
| Chikamori Hospital | 1 (2.3) | 139 (24.6) |  |  | 1 (3.7) | 3 (11.1) |  |
| Ebetsu City Hospital | 4 (9.1) | 30 (5.3) |  |  | 4 (14.8) | 0 (0.0) |  |
| Juzenkai Hospital | 0 (0.0) | 72 (12.7) |  |  | 0 (0.0) | 3 (11.1) |  |
| Kameda Medical Center | 39 (88.6) | 324 (57.3) |  |  | 22 (81.5) | 21 (77.8) |  |
| Treatment setting |  |  |  |  |  |  |  |
| Outpatient | 11 (25.0) | 111 (19.6) | 0.129 |  | 8 (29.6) | 10 (37.0) | 0.158 |
| Risk of bacterial resistance |  |  |  |  |  |  |  |
| Hospitalization for more than 2 days within 3 months | 4 (9.1) | 102 (18.1) | 0.264 |  | 4 (14.8) | 0 (0.0) | 0.590 |
| Residing in a nursing home or convalescent facility | 4 (9.1) | 126 (22.3) | 0.369 |  | 2 (7.4) | 4 (14.8) | 0.237 |
| Dialysis (within 30 days) | 0 (0.0) | 7 (1.2) | 0.158 |  | 0 (0.0) | 0 (0.0) | <0.001 |
| Comorbidity |  |  |  |  |  |  |  |
| Diabetes mellitus | 9 (20.5) | 138 (24.4) | 0.095 |  | 5 (18.5) | 5 (18.5) | <0.001 |
| Heart failure | 6 (13.6) | 69 (12.2) | 0.042 |  | 3 (11.1) | 6 (22.2) | 0.302 |
| Liver disease | 0 (0.0) | 26 (4.6) | 0.311 |  | 0 (0.0) | 0 (0.0) | <0.001 |
| Renal disease | 10 (22.7) | 68 (12.0) | 0.285 |  | 3 (11.1) | 3 (11.1) | <0.001 |
| Dementia | 3 (6.8) | 114 (20.2) | 0.399 |  | 1 (3.7) | 5 (18.5) | 0.485 |
| Malignancy | 6 (13.6) | 106 (18.8) | 0.139 |  | 5 (18.5) | 4 (14.8) | 0.100 |
| Asthma | 8 (18.2) | 65 (11.5) | 0.189 |  | 4 (14.8) | 5 (18.5) | 0.100 |
| COPD or bronchiectasis | 5 (11.4) | 154 (27.3) | 0.411 |  | 4 (14.8) | 4 (14.8) | <0.001 |
| Medication |  |  |  |  |  |  |  |
| Oral steroids | 5 (11.4) | 27 (4.8) | 0.244 |  | 2 (7.4) | 1 (3.7) | 0.162 |
| Antacids | 11 (25.0) | 188 (33.3) | 0.183 |  | 6 (22.2) | 6 (22.2) | <0.001 |
| Sleeping drugs | 2 (4.5) | 87 (15.4) | 0.368 |  | 2 (7.4) | 0 (0.0) | 0.400 |
| Aspiration-associated risk factors |  |  |  |  |  |  |  |
| Aspiration episodes | 4 (9.1) | 148 (26.2) | 0.460 |  | 2 (7.4) | 3 (11.1) | 0.128 |
| Pre-existing impaired consciousness | 3 (6.8) | 33 (5.8) | 0.040 |  | 1 (3.7) | 1 (3.7) | <0.001 |
| Neuromuscular diseases | 0 (0.0) | 50 (.8.8) | 0.441 |  | 0 (0.0) | 0 (0.0) | <0.001 |
| Insertion or placement of devices (e.g., nasogastric tubes) | 0 (0.0) | 15 (2.7) | 0.234 |  | 0 (0.0) | 0 (0.0) | <0.001 |
| Cerebrovascular diseases | 6 (13.6) | 138 (24.4) | 0.277 |  | 1 (3.7) | 2 (7.4) | 0.162 |
| Long-term bedridden status | 3 (6.8) | 86 (15.2) | 0.232 |  | 1 (3.7) | 2 (7.4) | 0.162 |
| Vital signs at diagnosis |  |  |  |  |  |  |  |
| Impaired consciousness | 6 (13.6) | 135 (23.9) | 0.265 |  | 2 (7.4) | 4 (14.8) | 0.237 |
| Heart rate, beats/minute | 96.14 (17.57) | 100.57 (19.96) | 0.236 |  | 94.04 (12.86) | 95.26 (26.78) | 0.058 |
| Respiratory rate, breaths/minute | 22.84 (4.78) | 23.17 (6.33) | 0.059 |  | 22.56 (5.10) | 21.11 (3.43) | 0.332 |
| Systolic blood pressure, mmHg | 127.80 (27.00) | 128.10 (25.05) | 0.012 |  | 129.15 (23.00) | 125.07 (16.68) | 0.203 |
| Body temperature, Celsius | 37.30 (1.16) | 37.55 (1.25) | 0.205 |  | 37.68 (1.16) | 37.31 (0.92) | 0.349 |
| Laboratory data at diagnosis |  |  |  |  |  |  |  |
| Hematocrit, % | 36.63 (3.75) | 37.02 (6.00) | 0.079 |  | 36.49 (3.31) | 37.80 (5.64) | 0.284 |
| BUN, mg/dL | 22.02 (14.62) | 24.18 (17.67) | 0.133 |  | 20.78 (10.55) | 17.90 (14.28)) | 0.229 |
| Na, mEq/L | 138.95 (3.04) | 137.78 (4.40) | 0.310 |  | 138.07 (2.66) | 138.67 (3.56) | 0.189 |
| Glucose, mg/dL | 139.07 (46.93) | 143.27 (60.40) | 0.078 |  | 146.24 (49.33) | 132.93 (56.20) | 0.252 |
| Albumin, g/dL | 3.32 (0.58) | 3.37 (0.54) | 0.084 |  | 3.33 (0.58) | 3.34 (0.42) | 0.022 |
| Pleural effusion on chest X-ray | 3 (6.8) | 40 (7.1) | 0.010 |  | 1 (3.7) | 1 (3.7) | <0.001 |
| CURB-65 |  |  |  |  |  |  |  |
| ≥3 | 10 (22.7) | 169 (29.9) | 0.164 |  | 4 (14.8) | 4 (14.8) | <0.001 |
| ≥4 | 5 (11.4) | 47 (8.3) | 0.102 |  | 3 (11.1) | 0 (0.0) | 0.500 |

Data are presented as number (%) or mean (standard deviation).

^a^Propensity score matching was conducted using the following 34 variables: age, sex, treatment setting (outpatient or inpatient), history of hospitalization (hospitalization for more than 2 d within 3 months before the diagnosis of CAP), residing in a nursing home or convalescent facility, dialysis (within 30 d before diagnosis), preexisting comorbidities (diabetes, heart failure, liver disease, renal disease, dementia, malignancy, asthma, and chronic respiratory disease [COPD and bronchiectasis]), prescribed drugs before admission (oral steroids, antacids, and sleeping drugs), aspiration-associated factors (aspiration episodes, pre-existing impaired consciousness, neuromuscular disease, insertion or placement of devices (e.g., nasogastric tubes), cerebrovascular disease, and long-term bedridden status), vital signs at diagnosis (consciousness, heart rate, respiratory rate, systolic blood pressure, and body temperature), laboratory data at diagnosis (hematocrit, BUN, sodium, glucose, and albumin), and findings of chest x-ray (pleural effusion). An SMD of <0.1 among the covariates was considered an appropriate match balance.

BL, beta-lactam monotherapy; BLM, beta-lactam plus macrolide; BUN, blood urea nitrogen; CAP; community-acquired pneumonia; COPD, chronic obstructive pulmonary disease; SMD, standardized mean difference

Supplementary Table S3 Primary and secondary endpoints for patients with CAP in the subgroup with microbiologically confirmed non-atypical bacterial pneumonia

|  | BLM (N = 25)^a^ | BL (N = 25)^a^ | Absolute difference |
| --- | --- | --- | --- |
| Primary endpoints |  |  |  |
| Death, % | 5.00 (0.00 – 15.38) | 6.45 (0.00–20.69) | 0.00 (−16.67 to 11.76) |
| Recovery, % | 93.75 (82.35–100.00) | 90.91 (75.00–100.00) | 3.57 (−12.50 to 20.00) |
| Secondary endpoints |  |  |  |
| Duration of antibiotic treatment (days) | 9.41 (8.21–10.67) | 9.36 (7.39–12.92) | 0.00 (−3.65 to 2.41) |
| Length of hospital stay (days)^b^ | 19.40 (12.00–30.45) | 19.00 (10.26–36.44) | 0.31 (−18.20 to 13.67) |

Values in parentheses indicate the 95% CI.

^a^N represents the point estimates derived from the median of the bootstrap results. The median and the 95% CI for N are 25 (15–37).

^b^Regarding the length of hospital stay, the number of cases was 12 (95%CI 4–22) in both the BLM and BL groups because these endpoints were assessed exclusively in hospitalized patients.

BLM, beta-lactam plus macrolide; BL, beta-lactam monotherapy; CI, confidence interval

Supplementary Table S4 Pretreatment variables of patients with CAP in the original dataset before and after propensity score matching, excluding patients with missing data

|  | Before matching | | |  | After matching | | |
| --- | --- | --- | --- | --- | --- | --- | --- |
| Variable | BLM  N = 146 | BL  N = 1482 | SMD^a^ |  | BLM  N = 131 | BL  N = 131 | SMD^a^ |
| Age, years | 67.86 (20.68) | 77.46 (13.88) | 0.545 |  | 70.67 (18.76) | 71.15 (16.63) | 0.027 |
| Female sex | 61 (41.8) | 577 (38.9) | 0.058 |  | 55 (42.0) | 49 (37.4) | 0.094 |
| Hospital |  |  | 0.991 |  |  |  | 0.922 |
| Chikamori Hospital | 4 (2.7) | 350 (23.6) |  |  | 3 (2.3) | 32 (24.4) |  |
| Ebetsu City Hospital | 15 (10.3) | 169 (11.4) |  |  | 15 (11.5) | 7 (5.3) |  |
| Juzenkai Hospital | 0 (0.0) | 202 (13.6) |  |  | 0 (0.0) | 13 (9.9) |  |
| Kameda Medical Center | 127 (87.0) | 761 (51.3) |  |  | 113 (86.3) | 79 (60.3) |  |
| Treatment setting |  |  |  |  |  |  |  |
| Outpatient | 41 (28.1) | 195 (13.2) | 0.375 |  | 31 (23.7) | 29 (22.1) | 0.036 |
| Risk of bacterial resistance |  |  |  |  |  |  |  |
| Hospitalization for more than 2 days within 3 months | 17 (11.6) | 290 (19.6) | 0.220 |  | 17 (13.0) | 13 (9.9) | 0.096 |
| Residing in a nursing home or convalescent facility | 10 (6.8) | 314 (21.2) | 0.422 |  | 10 (7.6) | 9 (6.9) | 0.029 |
| Dialysis (within 30 days) | 1 (0.7) | 23 (1.6) | 0.083 |  | 1 (0.6) | 3 (2.3) | 0.125 |
| Comorbidity |  |  |  |  |  |  |  |
| Diabetes mellitus | 32 (21.9) | 322 (21.7) | 0.005 |  | 30 (22.9) | 33 (25.2) | 0.054 |
| Heart failure | 16 (11.0) | 270 (18.2) | 0.207 |  | 16 (12.2) | 24 (18.3) | 0.170 |
| Liver disease | 2 (1.4) | 75 (5.1) | 0.210 |  | 2 (1.5) | 3 (2.3) | 0.056 |
| Renal disease | 12 (8.2) | 168 (11.3) | 0.105 |  | 11 (8.4) | 13 (9.9) | 0.053 |
| Dementia | 12 (8.2) | 281 (19.0) | 0.317 |  | 12 (9.2) | 12 (9.2) | <0.001 |
| Malignancy | 20 (13.7) | 281 (19.0) | 0.143 |  | 19 (14.5) | 23 (17.6) | 0.083 |
| Asthma | 9 (6.2) | 138 (9.3) | 0.118 |  | 9 (6.9) | 5 (3.8) | 0.136 |
| COPD or bronchiectasis | 21 (14.4) | 347 (23.4) | 0.232 |  | 21 (16.0) | 22 (16.8) | 0.021 |
| Medication |  |  |  |  |  |  |  |
| Oral steroids | 24 (16.4) | 102 (6.9) | 0.301 |  | 19 (14.5) | 22 (16.8) | 0.063 |
| Antacids | 42 (28.8) | 476 (32.1) | 0.073 |  | 39 (29.8) | 50 (38.2) | 0.178 |
| Sleeping drugs | 14 (9.6) | 198 (13.4) | 0.119 |  | 12 (9.2) | 13 (9.9) | 0.026 |
| Aspiration-associated risk factors |  |  |  |  |  |  |  |
| Aspiration episodes | 25 (17.1) | 441 (29.8) | 0.302 |  | 23 (17.6) | 14 (10.7) | 0.198 |
| Pre-existing impaired consciousness | 4 (2.7) | 100 (6.7) | 0.189 |  | 4 (3.1) | 7 (5.3) | 0.114 |
| Neuromuscular diseases | 6 (4.1) | 119 (8.0) | 0.165 |  | 6 (4.6) | 5 (3.8) | 0.038 |
| Insertion or placement of devices (e.g., nasogastric tubes) | 1 (0.7) | 36 (2.4) | 0.141 |  | 1 (0.8) | 1 (0.89) | <0.001 |
| Cerebrovascular diseases | 14 (9.6) | 400 (27.0) | 0.462 |  | 14 (10.7) | 13 (9.9) | 0.025 |
| Long-term bedridden status | 12 (8.2) | 205 (13.8) | 0.180 |  | 12 (9.2) | 10 (7.6) | 0.055 |
| Vital signs at diagnosis |  | | | | | | |
| Impaired consciousness | 15 (10.3) | 314 (21.2) | 0.303 |  | 15 (11.5) | 16 (12.2) | 0.024 |
| Heart rate, beats/minute | 96.71 (17.43) | 97.20 (20.06) | 0.026 |  | 97.01 (17.65) | 96.02 (16.71) | 0.057 |
| Respiratory rate, breaths/minute | 21.99 (5.23) | 22.77 (5.95) | 0.139 |  | 22.30 (5.24) | 22.54 (5.71) | 0.010 |
| Systolic blood pressure, mmHg | 129.14 (22.03) | 129.53 (24.69) | 0.016 |  | 129.82 (22.52) | 130.17 (22.14) | 0.016 |
| Body temperature, Celsius | 37.57 (1.02) | 37.48 (1.08) | 0.087 |  | 37.52 (0.98) | 37.50 (1.08) | 0.023 |
| Laboratory data at diagnosis |  |  |  |  |  |  |  |
| Hematocrit, % | 37.76 (5.00) | 36.60 (5.88) | 0.213 |  | 37.40 (5.01) | 37.51 (6.27) | 0.020 |
| BUN, mg/dL | 17.57 (11.70) | 22.74 (15.42) | 0.377 |  | 18.07 (12.10) | 20.35 (15.01) | 0.168 |
| Na, mEq/L | 137.82 (3.96) | 137.52 (4.94) | 0.065 |  | 137.77 (4.05) | 137.73 (4.43) | 0.009 |
| Glucose, mg/dL | 137.37 (52.03) | 140.02 (57.23) | 0.048 |  | 138.00 (52.43) | 140.50 (63.92) | 0.043 |
| Albumin, g/dL | 3.48 (0.58) | 3.39 (0.57) | 0.153 |  | 3.44 (0.58) | 3.51 (0.57) | 0.115 |
| Pleural effusion on chest X-ray | 2 (1.4) | 104 (7.0) | 0.285 |  | 2 (1.5) | 0 (0.0) | 0.176 |
| CURB-65 |  |  |  |  |  |  |  |
| ≥3 | 18 (12.3) | 365 (24.6) | 0.321 |  | 17 (13.0) | 23 (17.6) | 0.128 |
| ≥4 | 4 (2.7) | 87 (5.9) | 0.155 |  | 4 (3.1) | 7 (5.3) | 0.114 |

Data are presented as number (%) or mean (standard deviation).

^a^Propensity score matching was conducted using the following 34 variables: age, sex, treatment setting (outpatient or inpatient), history of hospitalization (hospitalization for more than 2 d within 3 months before the diagnosis of CAP), residing in a nursing home or convalescent facility, dialysis (within 30 d before diagnosis), preexisting comorbidities (diabetes, heart failure, liver disease, renal disease, dementia, malignancy, asthma, and chronic respiratory disease [COPD and bronchiectasis]), prescribed drugs before admission (oral steroids, antacids, and sleeping drugs), aspiration-associated factors (aspiration episodes, pre-existing impaired consciousness, neuromuscular disease, insertion or placement of devices (e.g., nasogastric tubes), cerebrovascular disease, and long-term bedridden status), vital signs at diagnosis (consciousness, heart rate, respiratory rate, systolic blood pressure, and body temperature), laboratory data at diagnosis (hematocrit, BUN, sodium, glucose, and albumin), and findings of chest x-ray (pleural effusion). An SMD of <0.1 among the covariates was considered an appropriate match balance.

BL, beta-lactam monotherapy; BLM, beta-lactam plus macrolide; BUN, blood urea nitrogen; CAP, community-acquired pneumonia; COPD, chronic obstructive pulmonary disease; SMD, standardized mean difference

Supplementary Table S5 Primary and secondary endpoints for patients with CAP in the complete case analysis

|  | BLM (N = 131) | BL (N = 131) | *P* |
| --- | --- | --- | --- |
| Primary endpoints |  |  |  |
| Death | 7 (5.3) | 11 (8.4) | 0.464 |
| Recovery | 121 (92.4) | 117 (89.3) | 0.521 |
| Secondary endpoints |  |  |  |
| Duration of antibiotic treatment (days) | 8.60 (4.34) | 9.27 (5.11) | 0.247 |
| Length of hospital stay^a^ (days) | 14.11 (16.29) | 16.73 (21.29) | 0.263 |

Data are presented as number (%) or mean (standard deviation).

^a^Regarding the length of hospital stay, the number of cases was 100 in the BLM group and 102 in the BL group because these endpoints were assessed exclusively in hospitalized patients.

BL, beta-lactam monotherapy; BLM, beta-lactam plus macrolide; CAP, community-acquired pneumonia

Supplementary Table S6 Pretreatment variables of patients with CAP in the subgroup matched with a caliper of 0.01, from one of the bootstrapped imputed datasets, before and after propensity score matching

|  | Before matching | | |  | After matching | | |
| --- | --- | --- | --- | --- | --- | --- | --- |
| Variables | BLM  N = 311 | BL  N = 2473 | SMD^a^ |  | BLM  N = 247 | BL  N = 247 | SMD^a^ |
| Age, years | 64.51 (20.63) | 76.04 (15.08) | 0.638 |  | 68.83 (18.22) | 70.49 (17.28) | 0.094 |
| Female sex | 142 (45.7) | 952 (38.5) | 0.145 |  | 109 (44.1) | 95 (38.5) | 0.115 |
| Hospital |  |  | 0.991 |  |  |  | 0.637 |
| Chikamori Hospital | 6 (1.9) | 484 (19.6) |  |  | 6 (2.4) | 30 (12.1 |  |
| Ebetsu City Hospital | 25 (8.0) | 344 (13.9) |  |  | 22 (8.9) | 32 (13.0) |  |
| Juzenkai Hospital | 0 (0.0) | 339 (13.7) |  |  | 0 (0.0) | 20 (8.1) |  |
| Kameda Medical Center | 280 (90.0) | 1306 (52.8) |  |  | 219 (88.7) | 165 (66.8) |  |
| Treatment setting |  |  |  |  |  |  |  |
| Outpatient | 134 (43.1) | 442 (17.9) | 0.570 |  | 84 (34.0) | 90 (36.4) | 0.051 |
| Risk of bacterial resistance |  |  |  |  |  |  |  |
| Hospitalization for more than 2 days within 3 months | 29 (9.3) | 500 (20.2) | 0.311 |  | 29 (11.7) | 24 (9.7) | 0.065 |
| Residing in a nursing home or convalescent facility | 15 (4.8) | 443 (17.9) | 0.421 |  | 15 (1.6) | 13 (5.3) | 0.035 |
| Dialysis (within 30 days) | 8 (2.6) | 40 (1.6) | 0.067 |  | 4 (1.6) | 8 (3.2) | 0.105 |
| Comorbidity |  |  |  |  |  |  |  |
| Diabetes mellitus | 51 (16.4) | 523 (21.1) | 0.122 |  | 48 (19.4) | 42 (17.0) | 0.063 |
| Heart failure | 27 (8.7) | 404 (16.3) | 0.233 |  | 27 (10.9) | 20 (8.1) | 0.097 |
| Liver disease | 4 (1.3) | 155 (6.3) | 0.264 |  | 4 (1.6) | 4 (1.6) | <0.001 |
| Renal disease | 27 (8.7) | 282 (11.4) | 0.091 |  | 20 (8.1) | 25 (10.1) | 0.070 |
| Dementia | 10 (3.2) | 424 (17.1) | 0.473 |  | 10 (4.0) | 9 (3.6) | 0.021 |
| Malignancy | 35 (11.8) | 510 (20.6) | 0.258 |  | 31 (12.6) | 33 (13.4) | 0.024 |
| Asthma | 26 (8.5) | 258 (10.4) | 0.132 |  | 17 (6.9) | 18 (7.3) | 0.016 |
| COPD or bronchiectasis | 50 (16.4) | 600 (24.3) | 0.306 |  | 39 (15.8) | 46 (7.7) | 0.075 |
| Medication |  |  |  |  |  |  |  |
| Oral steroids | 38 (12.5) | 201 (8.1) | 0.042 |  | 24 (9.7) | 19 (7.7) | 0.072 |
| Antacids | 82 (26.4) | 746 (30.2) | 0.084 |  | 66 (26.7) | 72 (29.1) | 0.054 |
| Sleeping drugs | 29 (9.3) | 315 (12.7) | 0.109 |  | 21 (8.5) | 16 (6.5) | 0.077 |
| Aspiration-associated risk factors |  |  |  |  |  |  |  |
| Aspiration episodes | 39 (12.5) | 708 (28.6) | 0.406 |  | 38 (15.4) | 33 (13.4) | 0.058 |
| Pre-existing impaired consciousness | 9 (2.9) | 159 (6.4) | 0.168 |  | 9 (3.6) | 2 (0.8) | 0.193 |
| Neuromuscular diseases | 8 (2.6) | 199 (8.0) | 0.246 |  | 8 (3.2) | 7 (2.8) | 0.024 |
| Insertion or placement of devices (e.g., nasogastric tubes) | 2 (0.6) | 64 (2.6) | 0.155 |  | 2 (0.8) | 2 (0.8) | <0.001 |
| Cerebrovascular diseases | 25 (8.0) | 608 (24.6) | 0.460 |  | 25 (10.1) | 26 (10.5) | 0.013 |
| Long-term bedridden status | 25 (8.0) | 332 (13.4) | 0.175 |  | 22 (8.9) | 15 (6.1) | 0.108 |
| Vital signs at diagnosis |  |  |  |  |  |  |  |
| Impaired consciousness | 30 (9.6) | 499 (20.2) | 0.299 |  | 24 (9.7) | 21 (8.5) | 0.042 |
| Heart rate, beats/minute | 97.26 (17.90) | 96.11 (20.39) | 0.060 |  | 95.82 (16.85) | 97.26 (19.29) | 0.080 |
| Respiratory rate, breaths/minute | 21.91 (5.57) | 22.52 (6.09) | 0.105 |  | 22.37 (5.53) | 22.43 (6.25) | 0.010 |
| Systolic blood pressure, mmHg | 127.08 (23.46) | 130.18 (25.79) | 0.126 |  | 128.64 (23.15) | 129.64 (24.21) | 0.042 |
| Body temperature, Celsius | 37.50 (1.06) | 37.45 (1.10) | 0.050 |  | 37.42 (1.09) | 37.45 (1.05) | 0.022 |
| Laboratory data at diagnosis |  |  |  |  |  |  |  |
| Hematocrit, % | 38.02 (5.46) | 36.59 (5.89) | 0.252 |  | 37.29 (5.65) | 37.72 (5.70) | 0.076 |
| BUN, mg/dL | 17.54 (12.99) | 22.39 (15.57) | 0.338 |  | 18.96 (14.00) | 18.77 (10.88) | 0.014 |
| Na, mEq/L | 137.80 (3.54) | 137.62 (4.64) | 0.044 |  | 137.58 (3.71) | 137.94 (4.04) | 0.093 |
| Glucose, mg/dL | 135.20 (59.95) | 139.67 (59.01) | 0.075 |  | 139.19 (64.91) | 140.44 (62.26) | 0.020 |
| Albumin, g/dL | 3.52 (0.55) | 3.41 (0.56) | 0.188 |  | 3.46 (0.56) | 3.47 (0.57) | 0.008 |
| Pleural effusion on chest X-ray | 11 (3.5) | 186 (7.5) | 0.175 |  | 11 (4.5) | 6 (2.4) | 0.111 |
| CURB-65 |  |  |  |  |  |  |  |
| ≥3 | 52 (16.7) | 621 (25.1) | 0.207 |  | 46 (18.6) | 46 (18.6) | <0.001 |
| ≥4 | 15 (4.8) | 129 (5.2) | 0.018 |  | 11 (4.5) | 11 (4.5) | <0.001 |

Data are presented as number (%) or mean (standard deviation).

^a^Propensity score matching was conducted using the following 34 variables: age, sex, treatment setting (outpatient or inpatient), history of hospitalization (hospitalization for more than 2 d within 3 months before the diagnosis of CAP), residing in a nursing home or convalescent facility, dialysis (within 30 d before diagnosis), preexisting comorbidities (diabetes, heart failure, liver disease, renal disease, dementia, malignancy, asthma, and chronic respiratory disease [COPD and bronchiectasis]), prescribed drugs before admission (oral steroids, antacids, and sleeping drugs), aspiration-associated factors (aspiration episodes, pre-existing impaired consciousness, neuromuscular disease, insertion or placement of devices (e.g., nasogastric tubes), cerebrovascular disease, and long-term bedridden status), vital signs at diagnosis (consciousness, heart rate, respiratory rate, systolic blood pressure, and body temperature), laboratory data at diagnosis (hematocrit, BUN, sodium, glucose, and albumin), and findings of chest x-ray (pleural effusion). An SMD of <0.1 among the covariates was considered an appropriate match balance.

BL, beta-lactam monotherapy; BLM, beta-lactam plus macrolide; BUN, blood urea nitrogen; CAP; community-acquired pneumonia; COPD, chronic obstructive pulmonary disease; SMD, standardized mean difference

Supplementary Table S7 Primary and secondary endpoints for patients with CAP in the subgroup matched with a caliper of 0.01

|  | BLM (N = 242)^a^ | BL (N = 242)^a^ | Absolute difference |
| --- | --- | --- | --- |
| Primary endpoints |  |  |  |
| Death, % | 5.56 (2.94–8.62) | 5.48 (2.58–9.05) | 0.00 (−4.29 to 4.21) |
| Recovery, % | 91.05 (87.24–94.33) | 91.25 (86.89–94.94) | 0.00 (−5.29 to 5.07) |
| Secondary endpoints |  |  |  |
| Duration of antibiotic treatment (days) | 9.96 (8.52–9.64) | 10.00 (9.02–18.32) | −0.97 (−9.35 to 0.20) |
| Length of hospital stay (days)^b^ | 17.83 (15.23–20.80) | 20.49 (17.22–24.67) | −2.68 (−7.49 to 1.71) |

Values in parentheses indicate the 95% CI.

^a^N represents the point estimates derived from the median of the bootstrap results. The median and the 95% CI for N are 242 (212–275).

^b^Regarding the length of hospital stay, the number of cases was 141 (95%CI 117–166) in both the BLM and BL groups because these endpoints were assessed exclusively in hospitalized patients.

BLM, beta-lactam plus macrolide; BL, beta-lactam monotherapy; CI, confidence interval
